# Incidence and factors associated with treatment failure among HIV infected adolescent and adult patients on second-line antiretroviral therapy in public hospitals of Northern Ethiopia: Multicenter retrospective study

**DOI:** 10.1371/journal.pone.0239191

**Published:** 2020-09-28

**Authors:** Adisu Zenebe Haftu, Abraham Aregay Desta, Nega Mamo Bezabih, Alemayehu Bayray Kahsay, Kibriti Mehari Kidane, Yodit Zewdie, Tewolde Wubayehu Woldearegay

**Affiliations:** 1 Abyi Adi Public General Hospital, Abyi Adi, Tigray, Ethiopia; 2 Tigray Health Research Institute, Mekelle, Tigray, Ethiopia; 3 School of Public Health, College of Health Sciences, Mekelle University, Mekelle, Tigray, Ethiopia; Ezintsha, a sub-division of Wits Reproductive Health and HIV Institute, SOUTH AFRICA

## Abstract

**Background:**

This study aimed to determine the incidence and factors associated with treatment failure among HIV infected adolescent and adult patients on second-line antiretroviral therapy (ART) in public hospitals of Northern Ethiopia.

**Methods:**

A retrospective study was conducted from September 1, 2007 to July 30, 2017 on 227 patients. The data were extracted using a retrieval checklist from the patient’s charts. The incidence rate of treatment failure was calculated using Kaplan–Meier methods and Cox proportional hazard model was used to assess factors associated with treatment failure.

**Result:**

The study subjects were followed for a total observation of 788.58 person-years with a median follow-up period of 35 (IQR: 17–60) months after switching to second-line ART. About 57 (25.11%) patients developed treatment failure, out of which, 32 (56.14%) occurred during the first two years. The overall incidence of second-line treatment failure was 72.3 per 1000 person years (95%CI: 55.75–93.71) of observation. The Kaplan–Meier estimates of cumulative treatment failure after 1, 2, and around 10 years of follow-up were 12.31% (95%CI: 8.60–17.45%), 14.99% (95%CI: 10.82%-20.57%), and 48.67% (95%CI: 32.45–67.81%) respectively. Age >45 years AHR = 3.33, 95%CI = 1.33–8.31), WHO stage IV (AHR = 3.63, 95%CI = 1.72–7.67), CD4 count <100 cells/mm^3^ (AHR = 3.79, 95%CI = 1.61–8.91), TB co-morbidity (AHR = 3.39 95%CI = 1.91–6.01) and poor adherence level (AHR = 3.63, 95% CI = 1.89–6.96) at the start of second line ART were significantly associated with second-line ART failure.

**Conclusion:**

Incidence of second-line ART treatment failure in the first 2 years of follow-up was high. The rate of second-line ART failure was higher in patients who started second-line ART with poor drug adherence, CD4 count <100 cells/mm^3^, TB co-morbidity, age >45 years, and being in WHO stage IV. Therefore, intensive counseling and adherence support should be given along with strong TB screening. Moreover, the government of Ethiopia should consider endorsing third-line ART drugs after careful cost–benefit analysis.

## Introduction

Out of the 37.9 million people living with HIV globally, 23.3 million were on treatment, more than three times as many as in 2010 [1]. The World Health Organization (WHO) estimates that 5% of the HIV infected patients were on second-line therapy by the end of 2015 [2]. The introduction of antiretroviral therapy (ART) has saved millions of lives by decreasing morbidity, mortality and number of new HIV infections [3, 4]. Although the burden of the epidemic continues and vary considerably between countries and regions, sub-Saharan Africa remains most affected, accounting for nearly two-thirds of the people living with HIV worldwide [5].

Ethiopia is among the high burden countries, with a prevalence of 1% and the 2018 estimate showed that about 690000 people were living with HIV/AIDS, 23000 new infections and 11000 people died from AIDS-related illness. Since 2010 the number of new HIV infections and deaths has decreased from 29000 to 23000 and from 20000 to 11000 respectively in 2018. About 66% of the adults and adolescents aged 15 years and above were on ART treatment in Ethiopia in 2018 [6]. According to data from PEPFAR annual program review for 2016, about 1.5% of the patients on ART in Ethiopia were transitioned to second-line ART [7].

The recommended first-line ART regimen in 2014 consists of nucleoside reverse transcriptase inhibitor (NRTI) backbone with one of the non-nucleoside reverse transcriptase inhibitor (NNRTI). Once-daily regimens comprising NRTI backbone (TDF + 3TC) and one NNRTI (EFV) are maintained as the preferred choices in adults, adolescents and children older than ten years. Boosted protease inhibitor (PI) + two NRTI combinations is recommended as the preferred strategy for second-line ART for adults, adolescents and also for children when NNRTI containing regimens were used in first-line ART [8]. The second-line ART regimens in Ethiopia were atazanavir/ritonavir or lopinavir/ritonavir based protease inhibitors. Patients who are switched to second-line ART after first-line treatment failure have improved outcomes. However, the proportion of patients failing on second-line regimen remains high. Patients on second-line tend to have overall worse adherence than patients who never fail [9, 10]. Approximately, 22%–23% of the patients on second-line ART in low-income countries experienced a virologic failure at 12 months after the start of second-line and mortality at 12 months ranges from 5.3%- 10.5% [9, 11]. It was also evidenced that there was a gradual rise in the number of patients failing, being on second-line ART regimens [12].

Even though ART reduces morbidity and mortality, non-adherence, drug resistance, and treatment failure are becoming a significant challenge to achieve better treatment outcomes. Antiretroviral treatment failure can be clinical, immunological or virological, which occurs when the ART regimen is unable to control viral replication [3]. The WHO adopted new recommendations for viral load (VL) testing as the preferred routine method to monitor patients on ART, but this may not be feasible in all settings. In low-and middle income countries, the WHO continues to recommend the use of CD4 and clinical monitoring to diagnose treatment failure and VL testing to confirm failure in order to avoid unnecessary changes in regimens [13]. Although VL is the gold standard method, clinical and immunological criteria are the main monitoring tools currently used for patient’s health status in Ethiopia [7]. These may result in late detection of treatment failure; which might compromise the durability and treatment outcome of second-line regimens.

Even though second-line ART was introduced in Ethiopia ten years prior, few studies have reported about the treatment outcome of second-line therapy. Although there were studies on treatment failure focusing on viral suppression and immunological failure on the failure of ART users [14, 15], none of these studies have addressed the effect of patient adherence on second-line ART treatment. To our knowledge, the factors contributing to treatment failure of HIV infected patients on second-line ART have not been investigated in the study area. To address this gap, this study was designed to investigate the incidence and factors associated with treatment failure in patients who were switched to second-line therapy at public hospitals of Northern Ethiopia. The findings of this study may be extrapolated to all patients on second-line ART regimen in Ethiopia.

## Methods and materials

### Study design, period and setting

A retrospective study was conducted from September 1, 2007 to July 30, 2017 in public hospitals of North Ethiopia (Ayder comprehensive specialized hospital (ACSH), Mekelle hospital and Quiha hospital) in Mekelle city, Tigray region, Northern Ethiopia. Mekelle is the capital city of Tigray Regional State which is situated 780 km away from the capital city of Ethiopia, Addis Ababa. The selected hospitals had the highest number of clients on second-line ART in the Tigray region. Since the start of ART, 9919 patients were enrolled on ART (204 on second-line ART) in Mekelle hospital. Quha hospital enrolled 796 patients on ART (27 on second-line ART) and Ayder comprehensive specialized hospital enrolled 1640 patients on ART (94 on second-line ART).

### Study population

All HIV infected patients who were switched to second-line ART from September 1, 2007 to July 30, 2017 in the selected hospitals of Northern Ethiopia.

### Inclusion criteria

- The disaggregation of PEPFAR was used and patients 15 years old and above who were on second-line ART for at least 6 months before treatment failure was diagnosed.
- Patients who were on care until January 31, 2018.

### Exclusion criteria

Patients who were on second-line ART but with unknown second-line start date and incomplete data on the basic variables were excluded from the study.

### Sample size calculation

The sample size required for Cox proportional hazard (PH) regression was calculated by using STATA statistical package, Version 14.0. Based on a previous study, assumptions of hazard-ratio (2.1) associated with second-line treatment failure, with a probability of event 0.423 [16], variability (standard deviation) of 0.494, 5% marginal error and 95% confidence interval with a power of 90% were used. These parameters were entered into the STATA software, the minimum sample size required for this study was estimated to be 185. However, since the source of the study population was small, all patients who fulfilled the inclusion criteria were included in the study.

### Sampling procedure

**Fig 1:**
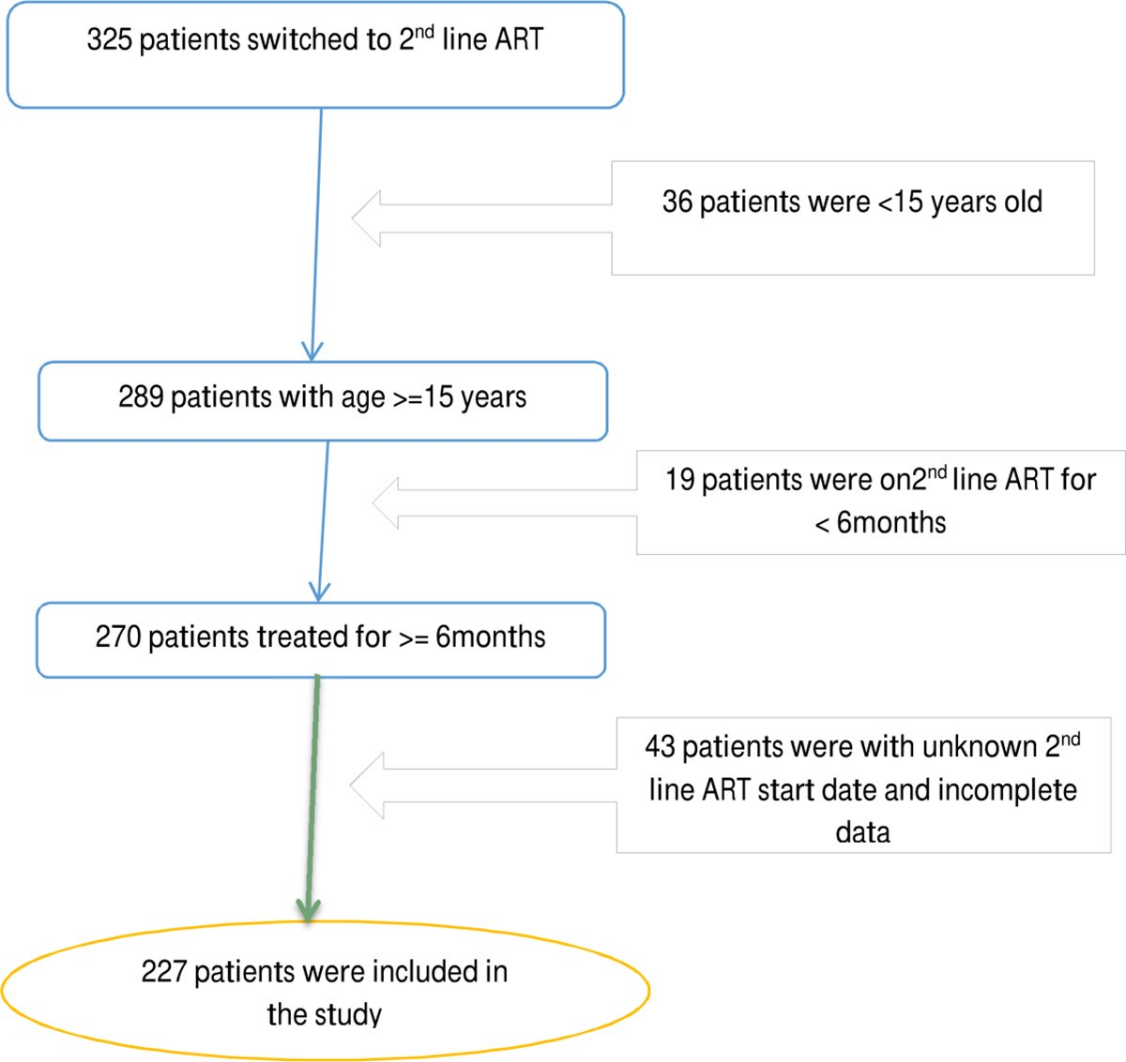
Schematic presentation of the sampling procedure of the study subjects.

There were 325 patients on second-line regimen in the selected hospitals. In total 227 patients (148 from Mekelle hospital, 61 from ACSH and 18 from Quiha hospital) who fulfilled the inclusion criteria were included in the study (Fig 1).

### Operational definitions

#### Clinical failure

New or recurrent clinical event indicating severe immunodeficiency (WHO clinical stage IV condition and certain WHO clinical stage III conditions (pulmonary tuberculosis (TB) and severe bacterial infections) after 6 months of effective treatment.

#### Immunologic failure

CD4 count falls to the baseline (or below) or persistent CD4 levels below 100 cells/mm^3^.

#### Virological failure

Plasma VL level above 1000 viral copies/ ml

#### Death

HIV/AIDS related death in patients who were on second-line ART

#### Censored

Patients who were transferred out, dropped out and any event out of the event of interest was considered as a censored.

#### Treatment failure

Includes clinical failure, immunological failure, virological failure and HIV/AIDS related death in patients who were on second-line ART. If a patient developed at least one of these four outcomes, he/she was considered as having treatment failure.

#### Poor adherence

Drug adherence of <85% or ≥6 ART drug doses missed out of 30 doses or >9 ART drug doses missed out of 60 doses.

#### Fair adherence

Drug adherence of 85–94% or 3–5 missed drug doses out of 30 doses or 3–9 missed drug doses out of 60 doses.

#### Good adherence

Drug adherence of 95% and above or ≤2 missed drug doses out of 30 doses or <3 missed drug doses out of 60 doses.

### Variables of the study

The independent variables of the study were selected from the health facilities’ ART follow-up clinic registration books, ART follow-up and patients’ charts.

### Dependent variable

The dependent variable was treatment failure to second-line ART which was defined as immunological failure, clinical failure, virological failure or HIV related death while on second-line ART. If a patient developed one of the four outcomes, he/she was considered to have a treatment failure [16–18].

### Independent variables

The independent variables were: age, CD4 count, and WHO clinical stage at the start of second-line ART. Additionally: sex, educational level, marital status, religion, residence, TB co-morbidity, number of first-line regimen changes, type of first-line regimen prior to switch, duration on first-line ART, second-line ART regimen and adherence level.

### Outcome measurements

The outcome of this study, i.e. treatment failure was assessed from the time of second-line ART initiation to immunological failure, clinical failure virological failure or HIV related death, which ever occurred first. Clinical failure was considered if a new or recurrent clinical event indicating severe immunodeficiency (WHO clinical stage IV condition and certain WHO clinical stage III conditions (pulmonary TB and severe bacterial infections)) after 6 months of effective treatment occurred. Immunological failure was considered if the CD4 count falls to the baseline (or below) or persistent CD4 levels below 100 cells/mm^3^ after at least 6 months on second-line ART. Virological failure was identified if plasma VL was above 1000 copies/ ml after at least 6 months on second-line ART. Finally, death was considered if the recorded death was due to HIV/AIDS in patients who were on second-line ART. The dependent variable was a binary outcome which was event (treatment failure) and censored (no treatment failure).

### Data collection tools/procedures

Secondary data were used for this study. The data was collected from the health facilities’ ART follow-up clinic charts and registration books, and patient cards by using retrieval check list S1–S3 Tables.

### Data quality control

The checklist was developed in English. Data were collected from the registration books and patient cards by four trained ART nurses who were in the ART clinics of the selected hospitals. The data collection procedure was closely supervised for assuring the quality of the data. The collected data were checked out for completeness, accuracy and clarity by taking samples.

### Data management and analysis

After the data were checked for its completeness and cleanness, it was entered and analyzed using STATA statistical software package, version 14.0. The frequency distribution of the variables was presented using tables. The incidence of treatment failure and cumulative probability of failure was calculated using Kaplan–Meier methods (S5 Fig). The factors associated with treatment failure were analyzed using Cox PH model. Bivariate Cox PH model were done at p-value of 0.05. Those variables with p-value ≤0.05 in the bivariate analysis were included in the multivariable Cox PH model analysis.

The multivariable Cox regression model development involved a series of backward selection procedure. Step by step exclusion of variables with large p-value was performed (S1 Fig). All possible interaction between the variables was checked by log likelihood. There was no any significant interaction in the model. Multicollinearity was checked using variance inflation factor (VIF). The mean VIF was 1.71 (less than 10), which indicates absence of multicollinearity between the variables (S3 Fig).

The Cox proportional assumption was tested based on Schoenfeld residuals (global test). Since the p-value of the global test was 0.1578 (> 0.05), the proportionality assumption of Cox PH model was met (S2 Fig). The fitness of the model was evaluated by using the Cox-Snell residuals. The plot of Nelson-Aalen cumulative hazard function versus Cox-Snell residuals were close to the straight line with some deviation at the upper left side of the graph. The deviation could be due to the presence of more censored data at the final follow-up time. Generally, the Cox model approximately fitted the data (S4 Fig). The 95% confidence interval (CI) of adjusted hazard ratio (AHR) was calculated and variables having a p-value of ≤ 0.05 in the multivariable Cox PH model were considered statistically significant (S1 Fig).

#### Ethics statement

Ethical clearance and approval was obtained from Mekelle University, College of Health Sciences, Research and Community Service Ethical Review Committee. Permission letter was obtained from Tigray regional health bureau and the respective hospitals. Nurses from the ART clinics of the selected hospitals were the data collectors to maintain privacy of the patients. The medical registration number (MRN) and unique ART number were used as unique identifiers. The data was not accessible by any other third party other than the study team. Informed consent was waived by the ethical review committee.

## Results

### Characteristics of the study participants

Almost half of the study participants, 118 (52%) were females. The median age of the patients at the start of second-line ART was 36 (inter quartile range (IQR): 30–43) years. Around half, 117 (51.5%) of the patients were married. Whereas, 51 (22.5%) and 35 (15.4%) were unmarried and divorced respectively. Most, 189 (83.3%) of the patients were urban dwellers. About, 52 (22.9%) of the patients had no formal education. The median duration of first-line ART before switching to second-line was 50 (IQR: 27–80) months. With regard to the number of regimen changes during first-line ART, 116 (51.1%) of the patients had no regimen change, 84 (37%) had regimen change once and 27 (11.9%) had regimen change twice. The frequently prescribed first-line NRTI backbones to patients at the initiation of second-line ART were: 113 (49.8%) on tenofovir disoproxil fumarate (TDF) + lamivudine (3TC), 82 (36.1%) on azidothymidine (AZT) + (3TC) and 28 (12.3%) were on stavudine (d4t) + (3TC) based regiments.

One hundred and seventy two (75.8%) of the patients were started on lopinavir (LP/r) boosted protease inhibitor second-line regimen and 55 (24.2%) received atazanavir (ATV/r) based regimen. Most of the patients, 198 (87.2%) had no TB. One hundred and sixty six patients (73.1%) had a CD4 count below 100 cells/mm^3^ at the start of second-line ART. At the start of second-line ART, 93 (41%) of the patients were in WHO clinical stage I or II, 85 (37.4%) were in WHO clinical stage III and 49 (21.6%) were in WHO clinical stage IV. With regard to the adherence level at the start of second-line ART, 193 (85%) of the patients had good or fair adherence level (Table 1).

**Table 1:** Socio-demographic and clinical characteristics of adolescent and adult patients on second-line ART at public hospitals of Northern Ethiopia, September 1, 2007 to July 30, 2017.

Variables		Censored	Failure (event)	Total	Bivariate analysis
		n = 170 (%)	n = 57 (%)	n = 227 (%)	p-value
Sex	Female	91 (53.5)	27 (47.4)	118 (52.0)	0.8201
	Male	79 (46.5)	30 (52.6)	109 (48.0)	
Age	15–29 yrs.	41 (24.1)	8 (14.0)	49 (22.0)	**0.0224**
	30–45 yrs.	104 (61.2)	36 (63.2)	140 (42.3)	
	>45 yrs.	25 (14.7)	13 (22.8)	38 (35.7)	
Religion	Muslim	7 (4.1)	5 (8.8)	12 (5.3)	0.1335
	Orthodox	161 (94.7)	52 (91.2)	213 (93.8)	
	Protestant	2 (1.2)	0	2 (0.9)	
Marital status	Never married	32 (18.8)	19 (33.3)	51 (22.5)	0.0593
	Married	90 (52.9)	27 (47.3)	117 (51.5)	
	Divorced	26 (15.3)	9 (15.8)	35 (15.4)	
	Separated	9 (5.3)	1(1.8)	10 (4.4)	
	Widowed	13 (7.7)	1 (1.8)	14 (6.2)	
Residence	Rural	30 (17.6)	8 (14.0)	38 (16.7)	0.8762
	Urban	140 (82.4)	49 (86.0)	189 (83.3)	
Educational level	No education	38 (22.4)	14 (24.6)	52 (22.9)	0.7911
	Primary	55 (32.3)	17 (29.8)	72 (31.7)	
	Secondary	64 (37.6)	19 (33.3)	83 (36.6)	
	Tertiary	13 (7.7)	7 (12.3)	20 (8.8)	
No. of regimen change during first-line ART	No change	94 (55.4)	22 (38.6)	116 (51.1)	**0.0014**
	One change	82 (48.3)	22 (38.6)	84 (37.0)	
	Two change	14 (8.3)	13 (22.8)	27 (11.9)	
NRTI backbone at switch*	d4t + 3TC	19 (11.2)	9 (15.8)	28 (12.3)	0.4954
	AZT + 3TC	63 (37.1)	19 (33.3)	82 (36.1)	
	TDF + 3TC	86 (50.5)	27 (47.4)	113 (49.8)	
	Other (ABC + ddl)	2 (1.2)	2 (3.5)	4 (1.8)	
Duration of first-line ART in years	<1	15 (8.8)	7 (12.3)	22 (9.7)	0.9879
	1–2	23 (13.5)	9 (15.8)	32 (14.1)	
	>2	132 (77.7)	41 (71.9)	173 (76.2)	
Second-line regimen	LPV/r based	124 (72.9)	48 (84.2)	172 (75.8)	0.5342
	ATV/r based	46 (27.1)	9 (15.8)	55 (24.2)	
Weight at switch*	< = 45 kg	60 (35.3)	23 (40.4)	83 (36.6)	0.1654
	>45kg	110 (64.7)	34 (59.6)	144 (63.4)	
TB status	No TB	161 (94.7)	37 (64.9)	198 (87.2)	**<0.001**
	TB present	9 (5.3)	20 (35.1)	29 (12.8)	
CD4 at switch*	<100cells/mm^3^	115 (67.6)	51 (89.5)	166 (73.1)	**0.0033**
	> = 100cells/mm^3^	55 (32.4)	6 (10.5)	61 (26.9)	
WHO stage at switch*	Stage I&II	81 (47.7)	12 (21.0)	93 (41.0)	**0.0001**
	Stage III	65 (38.2)	20 (35.1)	85 (37.4)	
	Stage IV	24 (14.1)	25 (43.9)	49 (21.6)	
Adherence at switch*	Good/fair (> = 85%)	150 (88.3)	43 (75.4)	193 (85.0)	**0.0096**
	Poor (<85%)	20 (11.7)	14 (24.6)	34 (15.0)	

*at switch: refers to “at the initiation of second line ART”.

**Abbreviations**: n, number; p-value, precession value; d4t, Stavudine; 3TC, lamivudine; AZT, azidothymidine; TDF, tenofovir disoproxil fumarate; ABC, abacavir; ddl, didanosine; LPV/R, Lopinavir/Ritonavir; ATV/r, Atazanavir/ritonavir; CD4, Cluster of Differentiation 4.

### Treatment failure to second-line ART

The median follow-up period on the patients was 35 (IQR: 17–60) months after switching to second-line ART with a total observation period of 788.58 person-years (PYs). A total of 57 patients developed a treatment failure, of which 32 (56.1%) occurred during the first two years of follow-up. Clinical failure, virological failure, HIV related death and immunological failure was used to diagnose 22, 14, 11 and 10 patients respectively. The overall incidence of second-line treatment failure was 72.3 per 1000 PYs (95% CI: 55.75–93.71) of observation. The incidence of treatment failure in the first two years of follow-up was 85.8 per 1000 PYs (95% CI: 60.70–121.30) but after two years, it was 60.10 per 1000 PYs **(**95% CI: 40.60–88.90).

Different rates of treatment failure were observed across different categories of variables. The rate of treatment failure was higher in patients who started second-line ART at age greater than 45 years compared to patients aged 15 to 29 years (136 vs. 37.6 per 1000 PYs). Higher incidence of treatment failure was observed in patients with TB co-morbidity compared to those without TB (258.10 vs. 52.03 per 1000 PYs). The rate of treatment failure was higher in patients who started on second-line ART at CD4 count <100 cells/mm^3^ compared to patients who were started at CD4 count ≥100 cells/mm^3^ (90.1 vs. 26.90 per 1000 PYs).

The incidence of treatment failure of patients in WHO clinical stage IV was higher compared to patients in WHO stage I or II at the initiation of second-line ART (142.01 vs. 38.2 per 1000 PYs). The incidence of treatment failure in patients with good/ fair and poor adherence levels were 62.4/1000 and 140.1/1000 PYs respectively (Table 2).

**Table 2:** Incidence of treatment failure among adolescent and adult patients on second-line ART at public hospitals of Northern Ethiopia, September 1, 2007 to July 30, 2017.

S. No.	Variable		Person-time	Failure n	Rate Per 1000 PYs	95% CI
1	Age	15–29	212.75	8	37.60	18.80–75.20
		30–45	480.67	34	74.90	54.02–103.83
		>45	95.17	15	136.00	79.30–235.20
2	TB status	No TB	711.08	37	52.03	37.70–71.80
		TB present	77.50	20	258.10	166.40–400.00
3	CD4 at switch	<100 cells/mm^3^	566.17	51	90.10	68.40–118.50
		≥100 cells/mm^3^	222.42	6	26.90	12.10–60.01
4	WHO clinical stage at switch	Stage I&II	314.25	12	38.20	21.60–67.20
		Stage III	298.30	20	67.00	43.20–103.90
		Stage IV	176.00	25	142.01	95.90–210.20
5	Adherence at switch	Good/ fair (≥85%)	689.00	43	62.41	47.20–85.80
		Poor (<85%)	99.58	14	140.10	83.20–237.30
	Overall incidence		788.58	57	72.30	55.75–93.71

**Abbreviations:** n, number of patients with treatment failure; PYs, person-years; CI, confidence interval.

**Fig 2:**
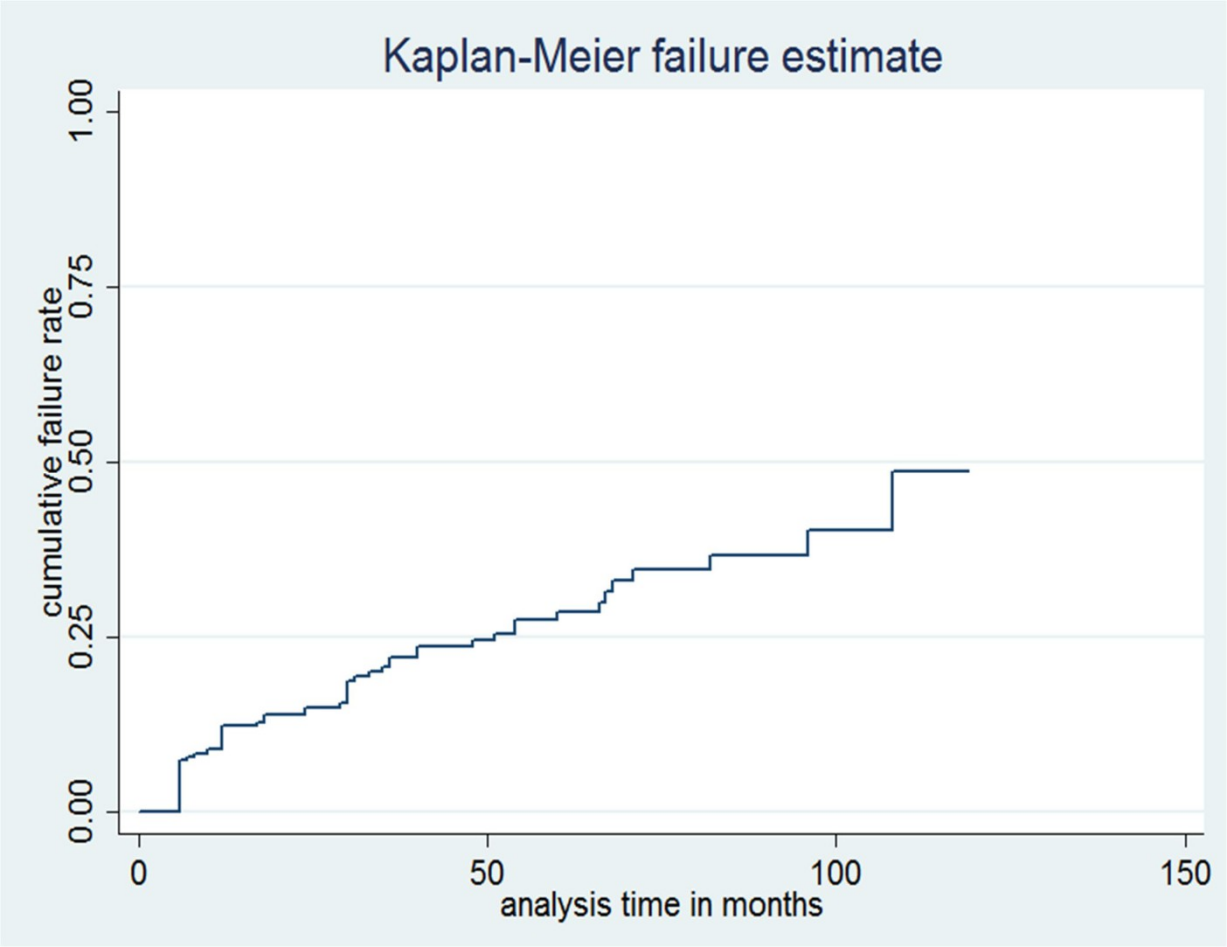
Kaplan-Meier failure curve showing hazard of treatment failure of adolescents and adult patients on second-line ART at public hospitals of Northern Ethiopia, September 1, 2007 to July 30, 2017.

The Kaplan–Meier estimates of cumulative treatment failure after 1, 2, 5 and around 10 years of follow-up were: 12.31% (95% CI: 8.60–17.45%), 14.99% (95% CI: 10.82%-20.57%), 28.65% (95% CI: 22.15%-36.58%) and 48.67% (95% CI: 32.45–67.81%) respectively (Table 3). The Kaplan-Meier estimate is shown across the months (Fig 2).

**Table 3:** Kaplan-Meier estimates of treatment failure among adolescent and adult patients on second-line ART at public hospitals of Northern Ethiopia, September 1, 2007 to July 30, 2017.

Time in months	Failure (n)	Cumulative failure (%)	95% CI
12	27	12.31	8.60–17.45
24	5	14.99	10.82–20.57
36	11	22.24	16.88–28.98
48	3	24.58	18.85–31.69
60	4	28.65	22.15–36.58
72	4	34.61	26.73–44.02
84	1	36.59	28.18–46.58
96	1	40.11	30.12–51.98
108	1	48.67	32.45–67.81
119	0	48.67	32.45–67.81

**Abbreviations**: n, number of patients with treatment failure; CI, confidence interval.

### Factors associated with second-line ART failure

#### Multivariable analysis

Variables which were found to be significant at p-value ≤ 0.05 in the final Cox PH model were age >45 years, TB co-morbidity, CD4 count <100 cells/mm^3^, WHO stage IV, and poor adherence at the start of second-line ART (Table 4).

**Table 4:** Multivariable Cox PH model analysis of factors associated with second-line ART failure among adolescent and adult patients at public hospitals of Northern Ethiopia, September 1, 2007 to July 30, 2017.

S. No.	Variable		Adjusted Hazard Ratio(AHR)	p-value	95% CI
1	Age	15–29 yrs.	Ref.		
		30–45 yrs.	2.14	0.056	0.98–4.71
		>45 yrs.	3.33	0.010	1.33–8.31
2	TB status	No TB	Ref.		
		TB present	3.39	<0.001	1.91–6.01
3	CD_4_ at switch*	≥100cells/mm^3^	Ref.		
		<100cells/mm^3^	3.79	0.002	1.61–8.91
4	WHO clinical stage at switch*	Stage I&II	Ref.		
		Stage III	1.41	0.354	0.67–2.96
		Stage IV	3.63	0.001	1.72–7.67
5	Adherence level at switch*	≥85% (Good/fair)	Ref.		
		<85% (Poor)	3.63	<0.001	1.89–6.96

*at switch: refers to “at the initiation of second-line ART”; Ref.: Reference.

#### Interpretation of the variables fitted to Cox PH model

Patients who started second-line ART at age >45 years were 3.3 times (AHR = 3.33, 95% CI = 1.33–8.31) more likely to develop treatment failure compared to patients in the age group of 15–29 years after adjusting for the effect of WHO stage, TB status, CD4 count and adherence. Patients who started second-line ART with advanced WHO stage (stage IV) had 3.6 times (AHR = 3.63, 95% CI = 1.72–7.67) higher likelihood of treatment failure compared to patients who started second-line ART at early WHO stages (stage I or II) keeping the other variables (age, TB status, CD4 count, adherence) constant.

The likelihood of second-line ART failure for patients with CD4 count below 100 cells/mm^3^ at the start of second-line ART was 3.8 times (AHR = 3.79, 95% CI = 1.61–8.91) higher compared to patients who started second-line ART at CD4 count of 100 cells/mm^3^ or above after controlling for the effect of age, TB status, WHO stage and adherence. Patients who started second-line ART with TB co-morbidity had 3.4 times (AHR = 3.39, 95% CI = 1.91–6.01) higher likelihood of treatment failure compared to patients without TB keeping the other variables constant. Patients with poor adherence level at start of second-line ART were 3.6 times (AHR = 3.63, 95% CI = 1.89–6.96) more likely to develop treatment failure compared to patients who had good/fair adherence level after adjusting for the effect of age, TB status, CD4 count and WHO stage (Table 4).

## Discussions

A retrospective study was conducted from September 1, 2007 to July 30, 2017 on 227 patients to determine incidence and factors associated with second-line antiretroviral treatment failure among adolescent and adult patients. The incidence of second-line treatment failure was 72.3 per 1000 PYs of observation. The cumulative probabilities of treatment failure at 1, 2, and around 10 years was 12.31%, 14.99%, and 48.67% respectively. More than half of the failures occurred during the first two years of follow-up. WHO stage IV, poor adherence at the start of second-line ART, CD4 count <100 cells/mm^3^ at the start of second-line ART, TB co-morbidity, and age >45 years were found to be significantly associated with second-line treatment failure.

The overall incidence of second-line treatment failure in this study was similar with studies conducted in African and Asian countries [18–20]. However, the incidence of the treatment failure of this study was lower as compared to the study conducted in South Africa, other studies from African and Asian patients, Amhara region, Ethiopia (9.86 per 100 PYs) and systematic analysis (15.0 per 100 PYs) [17, 21–23]. The cumulative incidence of this study is however higher compared to the study conducted in the north western Ethiopia (61.7/1000 PYs) [16]. The possible reason for this might be due to the difference in the duration of time taken for the studies. Since in most studies treatment failures on second-line ART occurred during the first year of follow-up period. A shorter follow-up period is likely to find a higher probability of failure when compared to a study with a longer follow-up period. Since this study used the recent WHO definition, difference in the diagnostic criteria used to identify treatment failure could be another possible reason. In those studies, VL was used mainly to assess treatment failure but due to limited access to VL in the current study, immunological and clinical criteria were mainly used to detect treatment failure. VL is the gold standard compared to the immunological and clinical markers which can shorten the time to diagnosis. Patients who have no immunological and clinical failure may already have virological failure and hence treatment failure.

In this study, CD4 count below 100 cells/mm^3^ at the start of second-line therapy was found to be significantly associated with second-line ART failure. Higher incidence of second-line treatment failure was observed in patients with CD4 count below 100 cells/mm^3^ as compared to those patients with 100 cells/mm^3^ or above at the start of second-line ART. This finding was consistent with studies conducted in the North West Ethiopia, Malawi, South Africa, and Vietnam [16–18, 24] which is partly due to the fact that patients with low CD4 (immune deficiency) have high probability of developing different opportunistic infections.

The incidence of treatment failure in patients with WHO stage IV was higher compared to patients with WHO stage I or II at the initiation of second-line ART. This finding is similar with the studies conducted in sub-Saharan Africa, Malawi and North West of Ethiopia [16, 25, 26]. Patients categorized in WHO clinical stage IV are likely to have advanced opportunistic infections and the drugs prescribed to treating opportunistic infection and antiretroviral drugs may interact. This minimizes the potency of second-line ART which may increase rate of treatment failure.

Concomitant TB infection was significantly associated with second-line ART treatment failure. This finding is similar with the studies conducted in South Africa and Uganda [11, 27, 28]. This is because TB itself could be an indication for treatment failure (clinical failure). The other possible explanation could be due to drug interactions between protease inhibitors and rifampicin. Rifampicin is a potent liver enzyme inducer, which significantly reduces the serum levels of protease inhibitors and concurrent use can lead to treatment failure.

Higher incidence of treatment failure was observed in patients with poor adherence as compared to patients with good/fair adherence level. This finding is consistent with the studies conducted in South Africa, Vietnam, resource limiting countries and Amhara region, Ethiopia [11, 17, 18, 22]. It is reasonable that strict adherence to ART plays a crucial role in the success of therapy for people living with HIV [29]. This is because patients with poor adherence to drug treatment may not take the drugs as prescribed by the physician. This can be justified, in the case of non-adherence, the level of antiretroviral drug concentration in the blood does not suffice to suppress the viral RNA replication and this in turn leads to second-line ART failure and emergence of drug-resistant viruses.

Higher incidence of treatment failure was observed in patients who started second-line ART at age greater than 45 years compared to patients between the age of 15 and 29 years. A finding from the study conducted in Asia showed that age above 40 years had higher second-line ART failure as compared to the those age <30 years [19]. But, a study conducted in Brazil reported younger age (<30 year) have been associated with increased second-line treatment failure [30]. This difference might be due to dynamic socio–demographic differences.

## Strengths and limitations of the study

This study has certain strengths. First, in comparison to the other studies, this study has used a long duration of follow-up of patients on second-line ART. Another strength was that the data was extracted from the routinely collected public health program system which can reflect the outcome of the current second-line ART management. Despite these strengths, the study misses some important variables like drug side effects during follow-up and body mass index and VL at start of second-line ART which were not recorded for most of the patients. Time to failure a first-line ART regimen and time to switch to second-line ART regimen after the first-line failure was identified was not included in the study. In addition, the immunological and clinical failure diagnostic criteria lacks both sensitivity and specificity to detect real treatment failure since the real treatment failure should be estimated by virological failure.

## Conclusions

Incidence of second-line ART treatment failure in the first 2 years of follow-up period was high. Age >45 years, WHO stage IV, poor adherence at the start of second-line ART, CD4 count <100 cells/mm^3^ and TB co-morbidity were found to be significantly associated with second-line treatment failure. Therefore, intensive counseling and adherence support should be given to patients with poor adherence along with strong TB screening. In addition, the government of Ethiopia should consider endorsing third-line ART drugs after careful cost–benefit analysis.

## Supporting information

S1 Fig STATA output multivariable cox -regression analysis of factors associated with second line ART. (PDF)

S2 Fig STATA output proportional assumption test. (PDF)

S3 Fig STATA output multicollinearity test using VIF. (PDF)

S4 Fig STATA output COX PH model goodness of fitness. (PDF)

S5 Fig STATA output incidence of treatment failure. (PDF)

S6 Fig STATA output cumulative probability treatment failure. (PDF)

S1 Table Data extraction checklist 1. (PDF)

S2 Table Data extraction checklist 2. (PDF)

S3 Table Data extraction checklist 3. (PDF)

S1 Text STROBE checklist. (DOCX)
