# Supplementary material for: Incidence and factors associated with treatment failure among HIV infected adolescent and adult patients on second-line antiretroviral therapy in public hospitals of Northern Ethiopia: Multicenter retrospective study
Source: PLoS One. 2020 Sep 28;15(9):e0239191. doi: 10.1371/journal.pone.0239191 (PMC7521713; doi:10.1371/journal.pone.0239191)
Supplement: S1 Fig — (PDF) [file pone.0239191.s001.pdf]

S1 Fig. STATA output multivariable cox -regression analysis of factors associated with second line ART

```
Cox regression -- Breslow method for ties

No. of subjects =          227                 Number of obs    =        227
No. of failures =           57
Time at risk    =         9463
                                               LR chi2(7)       =      61.84
Log likelihood  =   -246.98128                 Prob > chi2      =     0.0000

------------------------------------------------------------------------------
          _t | Haz. Ratio   Std. Err.      z    P>|z|     [95% Conf. Interval]
-------------+----------------------------------------------------------------
      agecat |
          2  |   2.149042   .8604591     1.91   0.056     .9804621    4.710415
          3  |   3.334325   1.554789     2.58   0.010     1.336885    8.316136
             |
  1.TBstatus |   3.393886   .9914678     4.18   0.000     1.914401    6.016747
2.adherenw~h |   3.629153    1.20781     3.87   0.000     1.890249    6.967734
      1.cCD4 |   3.788773    1.65298     3.05   0.002     1.611139     8.90972
             |
    whostage |
          2  |   1.416502   .5326737     0.93   0.354      .677835    2.960127
          3  |    3.63216   1.384478     3.38   0.001     1.720722    7.666891
------------------------------------------------------------------------------
```
