# Supplementary material for: Incidence and factors associated with treatment failure among HIV infected adolescent and adult patients on second-line antiretroviral therapy in public hospitals of Northern Ethiopia: Multicenter retrospective study
Source: PLoS One. 2020 Sep 28;15(9):e0239191. doi: 10.1371/journal.pone.0239191 (PMC7521713; doi:10.1371/journal.pone.0239191)
Supplement: S2 Fig — (PDF) [file pone.0239191.s002.pdf]

S2 Fig. STATA output proportional assumption test

```
. estat phtest

      Test of proportional-hazards assumption

      Time:  Time
```

	chi2	df	Prob>chi2
global test	10.58	7	0.1578
