# Supplementary material for: Incidence and factors associated with treatment failure among HIV infected adolescent and adult patients on second-line antiretroviral therapy in public hospitals of Northern Ethiopia: Multicenter retrospective study
Source: PLoS One. 2020 Sep 28;15(9):e0239191. doi: 10.1371/journal.pone.0239191 (PMC7521713; doi:10.1371/journal.pone.0239191)
Supplement: S3 Fig — (PDF) [file pone.0239191.s003.pdf]

S3 Fig. STATA output multicolinarity test using VIF

. vif, uncentered

Variable	VIF	1/VIF
agecat		
2	2.38	0.419793
3	1.33	0.752636
1.TBstatus	1.25	0.802574
2.adherenw~h	1.15	0.867156
1.cCD4	2.61	0.383190
whostage		
2	1.74	0.573211
3	1.53	0.653590
Mean VIF	1.71	
