# Supplementary figures and images for: Incidence and factors associated with treatment failure among HIV infected adolescent and adult patients on second-line antiretroviral therapy in public hospitals of Northern Ethiopia: Multicenter retrospective study
Source: PLoS One. 2020 Sep 28;15(9):e0239191. doi: 10.1371/journal.pone.0239191 (PMC7521713; doi:10.1371/journal.pone.0239191)

**S4 Fig. STATA output COX PH Model Goodness of fitness**

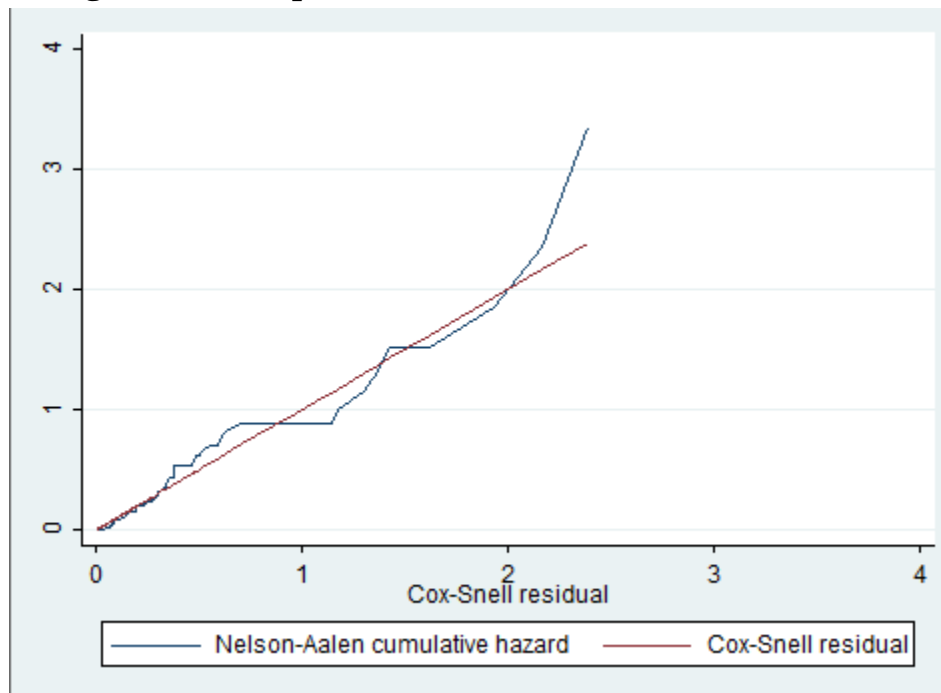

Supplement: S4 Fig — (PDF) [file pone.0239191.s004.pdf]
