# Supplementary material for: Incidence and factors associated with treatment failure among HIV infected adolescent and adult patients on second-line antiretroviral therapy in public hospitals of Northern Ethiopia: Multicenter retrospective study
Source: PLoS One. 2020 Sep 28;15(9):e0239191. doi: 10.1371/journal.pone.0239191 (PMC7521713; doi:10.1371/journal.pone.0239191)
Supplement: S5 Fig — (PDF) [file pone.0239191.s005.pdf]

S5 Fig. STATA output incidence of treatment failure

```
. stptime

         failure _d:  ttoutcome == 1
   analysis time _t:  timeyrs
                 id:  id
```

Cohort	person-time	failures	rate	[95% Conf.	Interval]
total	788.58333	57	.07228152	.0557549	.0937069

.

adheren~h	person-time	failures	rate	[95% Conf.	Interval]
>=85%(g~)	689	43	.06240929	.0462852	.0841504
<85%(po~)	99.583333	14	.14058577	.0832623	.2373748

whostage	person-time	failures	rate	[95% Conf.	Interval]
stage1&2	314.25	12	.03818616	.0216863	.0672398
stage-3	298.33333	20	.06703911	.0432508	.1039113
stage-4	176	25	.14204545	.0959814	.2102169

TBstatus	person-time	failures	rate	[95% Conf.	Interval]
no TB	711.08333	37	.05203328	.0377003	.0718155
TBpresent	77.5	20	.25806452	.1664922	.4000025

cCD4	person-time	failures	rate	[95% Conf.	Interval]
<100cel~3	566.16667	51	.09007948	.0684595	.1185272
>=100ce~3	222.41667	6	.0269764	.0121194	.0600462

agecat	person-time	failures	rate	[95% Conf.	Interval]
15-29	212.75	8	.03760282	.0188051	.075191
30-45	480.66667	36	.07489598	.0540246	.1038306
>45	95.166667	13	.13660245	.0793191	.2352553

```
. stptime, at(2)

         failure _d:  ttoutcome == 1
   analysis time _t:  timeyrs
                 id:  id
```

Cohort	person-time	failures	rate	[95% Conf.	Interval]
(0 - 2]	372.75	32	.08584842	.0607099	.1213962
> 2	415.83333	25	.06012024	.0406238	.0889736
total	788.58333	57	.07228152	.0557549	.0937069
