# Supplementary material for: Incidence and factors associated with treatment failure among HIV infected adolescent and adult patients on second-line antiretroviral therapy in public hospitals of Northern Ethiopia: Multicenter retrospective study
Source: PLoS One. 2020 Sep 28;15(9):e0239191. doi: 10.1371/journal.pone.0239191 (PMC7521713; doi:10.1371/journal.pone.0239191)
Supplement: S6 Fig — (PDF) [file pone.0239191.s006.pdf]

S6 Fig. STATA output cumulative Probability treatment failure

```
. sts list, failure at(12 24 36 48 60 72 84 96 108 119)

         failure _d:  ttoutcome == 1
   analysis time _t:  time
```

Time	Beg. Total	Fail	Failure Function	Std. Error	[95% Conf. Int.]	
12	187	27	0.1231	0.0222	0.0860	0.1745
24	151	5	0.1499	0.0246	0.1082	0.2057
36	112	11	0.2224	0.0307	0.1688	0.2898
48	90	3	0.2458	0.0327	0.1885	0.3169
60	62	4	0.2865	0.0368	0.2215	0.3658
72	40	4	0.3461	0.0442	0.2673	0.4402
84	26	1	0.3659	0.0471	0.2818	0.4658
96	18	1	0.4011	0.0561	0.3012	0.5198
108	7	1	0.4867	0.0927	0.3245	0.6781
119	1	0	0.4867	0.0927	0.3245	0.6781

Note: failure function is calculated over full data and evaluated at indicated times; it is not calculated from aggregates shown at left.
