# Supplementary material for: Incidence and factors associated with treatment failure among HIV infected adolescent and adult patients on second-line antiretroviral therapy in public hospitals of Northern Ethiopia: Multicenter retrospective study
Source: PLoS One. 2020 Sep 28;15(9):e0239191. doi: 10.1371/journal.pone.0239191 (PMC7521713; doi:10.1371/journal.pone.0239191)
Supplement: S1 Table — (PDF) [file pone.0239191.s007.pdf]

S1 Table. Data extraction checklist 1

1. **Name of health facility…………………………..facility code………**

s.no	**socio demographic characteristics**		
	**Questions**	**Answer**	**remark**
201	Sex	1. Male 2. female	
202	Age(years)	…………..	
203	Patient's residence	1. Urban 2. rural	
204	Marital Status	1. Never married 2. Married<br>3. Divorced 5. Widowed<br>6. separated	
205	Level of education	1. No education 2. Primary<br>3. Secondary 4.Tertiary	
206	Religion	1. Muslim 2. Orthodox 3. Protestant<br>4. Catholic 5.Other specify…………	
207	Occupation	1 Recorded (Specify)………………..<br>2 Not recorded ………….	
	**HIV Care and ART Information**		
	**Questions**	**answer**	**Remark**
301	Study identification number.	………………………………	
302	Date confirmed HIV positive	………../……/……..	
303	Date of eligibility	………../……/……..	
304	Reason for eligibility for ART	1. Clinical only 2. CD4 only 3. VL only<br>4. Clinical&CD4 5.CD4&VL 6. Clinical& VL<br>7. Other (TB, Pregnancy,)	
305	Date of first ART start	………../……/……..	
306	Number of regiment change during first line ART	………………………………………	
307	First line ART regiment during switch to 2nd line ART	………………………….	

308	Date of second line ART start	………../……/……..	
309	Criteria for switch to second line:	1. Clinical Only 2.CD4 only 3.VL only<br>4. Clinical and CD4 5. CD4 and VL<br>6.clinical and VL 7. Clinical, CD4&VL	
310	Duration of first line ART uses in months	…………….months	
