# Supplementary material for: Incidence and factors associated with treatment failure among HIV infected adolescent and adult patients on second-line antiretroviral therapy in public hospitals of Northern Ethiopia: Multicenter retrospective study
Source: PLoS One. 2020 Sep 28;15(9):e0239191. doi: 10.1371/journal.pone.0239191 (PMC7521713; doi:10.1371/journal.pone.0239191)
Supplement: S2 Table — (PDF) [file pone.0239191.s008.pdf]

S2 Table. Data extraction checklist 2

**Parameters during and after second line ART start**																
	date															
	**parameters**	At switch to 2nd line	Month 6	Month 12	Month 18	Month 24	Month 30	Month 36	Month 42	Month 48	Month 54	moth 60	Moth 66	Moth 72	Month 84	Month ≥96
401	Weight (kg)															
402	BMI															
403	WHO staging(1-4)															
404	TB(yes or no)															
405	2nd line Drug regimen(name)															
406	CD4 count															
407	Viral load test															
	**ADHERENCE**															
408	Adherence status (good, Fair, poor)															

409	Drug Sideeffect<br><br>(yes or no)															
