# Supplementary material for: Incidence and factors associated with treatment failure among HIV infected adolescent and adult patients on second-line antiretroviral therapy in public hospitals of Northern Ethiopia: Multicenter retrospective study
Source: PLoS One. 2020 Sep 28;15(9):e0239191. doi: 10.1371/journal.pone.0239191 (PMC7521713; doi:10.1371/journal.pone.0239191)
Supplement: S3 Table — (PDF) [file pone.0239191.s009.pdf]

S3 Table. Data extraction checklist 3

**follow-up events**			
	Question	Answer	remark
501	End of follow-up date	……………/……../………..	
501	Event(outcome)	1. clinical failure<br>2. virological failure<br>3. Immunological failure<br>4. Death due to HIV/AIDS	
502	Patient category	1. Alive<br>2. transfer out<br>3. dropped(lost from the program)	
502	Time to treatment failure(months)		
